# Emotional Vocalizations Cause Auditory Distraction That Affects Word Repetition and Behavioral Listening Effort

**DOI:** 10.1097/AUD.0000000000001834

**Published:** 2026-05-08

**Authors:** Erin M. Picou, Shae Morgan, Steven C. Marcrum, Elizabeth D. Young, Travis M. Moore, Samantha J. Gustafson

**Affiliations:** 1 Department of Hearing and Speech Sciences, Vanderbilt University Medical Center, Nashville, Tennessee, USA; 2 Program in Audiology, University of Louisville, Louisville, Kentucky, USA; 3 Department of Otolaryngology, University Hospital Regensburg, Regensburg, Germany; 4 Department of Communication Sciences and Disorders, University of Utah, Salt Lake City, Utah, USA; 5 Accenture, Eden Prairie, Minnesota, USA; 6 Department of Speech, Language and Hearing Sciences, Indiana University, Bloomington, Indiana, USA.

**Keywords:** Background noise, Distraction, Emotion, Listening effort, Valence

## Abstract

**Objectives:**

Task-irrelevant sounds can be distracting, impairing speech recognition and increasing listening effort. One way to measure distraction is to evaluate performance differences between trials that do and do not contain task-irrelevant sounds. Poorer performance following irrelevant sounds is interpreted as evidence of distraction. Using this general approach, previous work has demonstrated that the degree to which irrelevant non-speech sounds are distracting is related to stimulus valence. That is, unpleasant and pleasant sounds are more distracting than neutral ones. The purpose of the present study was to evaluate the effect of the presence of task-irrelevant, human vocalizations on word repetition performance and behavioral listening effort.

**Design:**

Eighteen adults with normal hearing participated in a dual-task paradigm where the primary task was word repetition, and the secondary task was word categorization speed, with speed being interpreted as a measure of behavioral listening effort. Immediately before 45% of the target words, a task-irrelevant vocalization was presented. Each list contained trials with and without irrelevant vocalizations; the difference in performance between trial types was interpreted as distraction. Conditions varied by background noise (quiet, noise) and by valence of the vocalization (pleasant, unpleasant, neutral). Conditions were blocked such that testing with each list of words was completed with a single combination of noise and valence vocalization. Data were analyzed using linear mixed-effects modeling to evaluate the effect of task-irrelevant vocalization presence, vocalization valence, and background noise on word repetition performance and behavioral listening effort.

**Results:**

As expected, background noise negatively affected word repetition performance (~6 percentage points) and increased behavioral listening effort (119 msec slowing of secondary task response times). The valence of the task-irrelevant sound within a condition did not affect word repetition performance, but did affect behavioral listening effort. Response times were longer (~40 msec longer) during the conditions with pleasant or unpleasant vocalizations than during conditions when neutral vocalizations were interleaved with target words. No distraction was noted in word recognition performance; performance was similar for trials with and without task-irrelevant vocalizations. Distraction effects were evident in behavioral listening effort; response times were ~30 msec longer in trials preceded by task-irrelevant vocalizations than in trials without task-irrelevant vocalizations. The calculated distraction effects were similar across conditions.

**Conclusions:**

Although the valence of the vocalizations generally increased listening effort, slowing secondary task times in conditions with valenced task-irrelevant vocalizations, the calculated distraction effects within a condition did not depend on valence or the presence of background noise. Moreover, the task-irrelevant vocalizations increased behavioral listening effort in quiet and in noise, despite minimal changes in word repetition performance. Future work is warranted to disentangle the methodological differences and the differences in effect sizes between the present study and previous work with non-speech sounds. The findings have implications for listening in complex, real-world environments, where distraction and valenced sounds are likely to co-occur.

## INTRODUCTION

Listening to and understanding speech can be modeled as a combination of bottom-up and top-down processes, dependent upon both the acoustic properties of the incoming signals and on the cognition required to recognize and interpret the speech signal (Beutelmann et al. 2010; Rönnberg et al. 2013; Pichora-Fuller et al. 2016). While it is clear that the acoustic characteristics of a speech signal and environmental maskers can affect speech perception (Rhebergen et al. 2006; Beutelmann et al. 2010), the impact of cognitive factors is also important. For example, the role of attention has long been implicated in the ability to segregate target speech from other auditory objects (Bregman 1990; Shinn-Cunningham & Best 2008). When the speech signal of interest (or other auditory object) changes (e.g., a switch in communication partners), a listener directs their attention to the new target and must reset the perceptual grouping to segregate the acoustic stream (Cusack et al. 2004; Ben-David et al. 2012; Maddox et al. 2012). The efficiency and accuracy with which the listener performs this cognitive task could affect speech perception performance in acoustically complex environments.

Shifts toward a new auditory object might not always be voluntary or desirable (e.g., hearing a barking dog during a conversation). In laboratory settings, task-irrelevant stimuli have been shown to impair speech recognition performance (Gustafson et al. 2023; Morgan et al. 2025). For example, Gustafson et al. (2023) studied the potential distraction effect by presenting task-irrelevant, non-speech sounds before 24% of target words during a word repetition task in quiet and in noise. The participants, adults with normal hearing, demonstrated reduced word repetition performance for words that immediately followed task-irrelevant sounds, but only in quiet. The distraction effect on word repetition was not evident in the presence of background noise. This finding is consistent with the Load Theory of Attention (Lavie & Tsal 1994; Lavie 1995). The Load Theory of Attention states that distraction is most likely to occur when the perceptual load of the task is low. In conditions of low perceptual load (i.e., in quiet), listeners have unused cognitive resources that are available and are susceptible to distraction. Under conditions of higher perceptual load (i.e., in noise), listeners have fewer resources available to be captured by the task-irrelevant stimuli.

Unlike word repetition performance, where distraction effects depended on the absence of background noise, Gustafson et al. (2023) reported distraction effects on verbal response times in quiet and in background noise. Specifically, they found that verbal response times were slower for words that followed task-irrelevant, non-speech sounds than for words that did not follow task-irrelevant sounds when words were presented either in quiet or in noise. This finding was interpreted as increased listening effort due to auditory distraction. Here, listening effort is described as the cognitive resources necessary to understand speech (Hicks & Tharpe 2002; Rönnberg et al. 2013; Picou et al. 2017) and reflects the cognitive cost of speech processing and recognition. The finding that task-irrelevant sounds increase listening effort lends support to existing models of effort, where listening effort occurs as the result of both bottom-up (e.g., masking, reverberation) and top-down processes (e.g., arousal, motivation, attention; Rönnberg et al. 2013; Pichora-Fuller et al. 2016). In summary, existing work demonstrates that task-irrelevant, non-speech sounds can impair speech repetition performance in quiet and can increase behavioral listening effort in quiet and in noise.

One limitation in the existing work is the exclusive use of non-speech sounds (e.g., animals barking, music, doorbells) as task-irrelevant stimuli (Gustafson et al. 2023; Morgan et al. 2025). Not all potentially distracting sounds in the environment will be non-speech sounds; irrelevant sounds could be vocalizations (e.g., laughter, groaning). Human vocalizations, which are social in nature, are processed differently in the auditory cortex than nonsocial, non-speech sounds (Peretz & Coltheart 2003; Meyer et al. 2005; Lima et al. 2013). Therefore, speech sounds might recruit different areas of the cortex than non-social sounds and, by extension, could have differential effects on distractibility than non-speech sounds.

Previous research provides inconsistent support for this hypothesis. Some investigators have reported that auditory distraction effects on memory tasks are greater for meaningful speech than for non-meaningful speech (Marsh et al. 2009), greater for meaningful speech than for noise (Jones et al. 1990), and greater for meaningful speech than for environmental sounds (Leist et al. 2025). Others have found that speech sounds are less distracting than non-speech sounds (Reiche et al. 2013) or just as distracting as non-speech sounds (Jones & Macken 1993) when studying involuntary attentional shifts and visual serial recall tasks, respectively. Therefore, it is not clear if human vocalizations would yield similar distraction effects as the non-speech sounds used by Gustafson et al. (2023).

It is important to note that the degree to which vocalizations are distracting in the laboratory might also be dependent on the emotion of the task-irrelevant stimuli. Within the dimensional view, emotions can be described along the combination of several continua, often valence and arousal (Osgood et al. 1957; Russell 1980; Bradley & Lang 1994); valence reflects hedonistic valence (i.e., the degree to which an emotion is pleasant or unpleasant), and arousal reflects the degree of activation of an emotion. For distraction effects, the valence of the task-irrelevant stimulus has been shown to affect the degree to which the stimulus is distracting in the visual domain (Bradley et al. 2012) and the auditory domain (Morgan et al. 2025). Specifically, in a secondary analysis of the data reported by Gustafson et al. (2023), Morgan et al. (2025) found that both pleasant and unpleasant sounds were more distracting than neutral ones in terms of word repetition performance (in quiet) and in terms of verbal response times (in quiet and in noise). In contrast, Morgan et al. found no effect of stimulus arousal category on distraction. Therefore, auditory distraction was dependent on the emotional valence content of the distractor when it was a non-speech sound; however, it is unknown if the same is also true when the task-irrelevant sound is speech.

### Purpose

The primary purpose of this study was to evaluate the effect of task-irrelevant human vocalizations on word repetition performance and behavioral listening effort (secondary task response times), using a dual-task paradigm. Task-irrelevant vocalizations were recordings of humans producing a vowel sound while conveying pleasant, neutral, or unpleasant emotions. Differences in word repetition or listening effort between trials with and without task-irrelevant vocalizations were interpreted as evidence of distraction. To avoid introducing distraction based on semantic or informational content, task-irrelevant vocalizations did not contain phonologically meaningful speech. It was hypothesized that the presence of pleasant and unpleasant task-irrelevant vocalizations would be distracting and reduce word repetition performance, but only in quiet (low cognitive load) and not in noise (higher cognitive load). It was also expected that distraction effects would be evident for listening effort in quiet and in noise, but the effects would be largest for valenced (pleasant and unpleasant) vocalizations compared with neutral vocalizations. This pattern of results would support previous findings (Gustafson et al. 2023; Morgan et al. 2025) and extend the conclusions in previous studies with non-speech, task-irrelevant sounds to task-irrelevant human vocalizations.

## MATERIALS AND METHODS

Participants completed a dual-task paradigm to simultaneously measure word repetition performance (primary task) and behavioral listening effort (secondary task; response times). During the dual-task testing, task-irrelevant vocalizations were presented immediately before the target word on a pre-determined proportion of trials for each list. Trials with and without task-irrelevant vocalizations were compared in order to evaluate the effect of the task-irrelevant vocalization’s valence on word repetition performance and behavioral listening effort. That is, worse word repetition performance or longer response times when task-irrelevant vocalizations were present were interpreted as evidence of distraction for word repetition and behavioral listening effort, respectively. Participants also completed subjective ratings to indicate their emotional state immediately following each list (these data are not presented in this paper).

### Participants

Eighteen adults (22 to 47 years old, *M* = 27.2, SD = 5.8) with self-reported normal hearing were recruited to participate. Before testing, all participants passed an air conduction hearing screening at 20 dB HL in each ear at octave frequencies between 250 and 8000 Hz in an audiometric test booth. Participants identified as either male (n = 4) or female (n = 14). Two additional adults (25- and 23-year-old females) were recruited and began the study, but withdrew citing time constraints. All participants reported normal development and cognitive function. Participants denied otologic symptoms, such as pain, otorrhea, and vertigo. Testing occurred over the course of two visits, and participants were paid on a per-session basis. The study was conducted with approval from the Institutional Review Board at the first author’s institution.

### Stimuli

#### Dual-Task Paradigm

The target words for the dual-task paradigm were developed previously (Picou et al. 2011, 2014) and have been used extensively in laboratory measures of listening effort (Picou et al. 2016, 2017; Picou & Ricketts 2017, 2018; Strand et al. 2018; Huang et al. 2022). Briefly, the words used in the primary task were monosyllables spoken by a female talker with a general American accent, scaled to all have the same approximate root mean square level. The words were arranged into eight lists of 60 words, each with approximately equal intelligibility based on pilot testing during stimulus development. Background noise was a four-talker babble with female talkers, developed previously for this paradigm (Picou et al. 2011, 2014).

#### Task-Irrelevant Vocalizations

The task-irrelevant vocalizations were from the Montreal Affective Voices (MAV) corpus (Belin et al. 2008). The MAV corpus includes brief affective vocalizations in nine emotions (angry, disgusted, fearful, happy, neutral, painful, pleased, sad, and surprised), each portrayed by actors while speaking the vowel /æ/ (as in apple). There were 10 actors (male and female) represented in the published MAV corpus. The MAV stimuli were validated during development (Belin et al. 2008) and have been used extensively in the study of auditory emotion perception (Vasconcelos et al. 2017; Lausen & Schacht 2018; Oliva & Anikin 2018).

To be consistent with previous work in this area (Morgan et al. 2025), vocalizations were chosen to reflect three categories of emotion valence—pleasant, neutral, and unpleasant. The pleasant valence was represented by “happiness” and “pleasure.” The neutral valence was represented by the “neutral” recordings. The negative valence was represented by the emotions of “sadness,” “anger,” “surprise,” and “pain.” For each valence category, nine MAV sounds were chosen and represented by nine talkers.* Table 1 shows the ratings of valence of the tokens used in this study for each emotion, as reported by Belin et al. (2008). Note that the sounds in the “pleasant” category generally elicited high ratings of valence, whereas the sounds in the “unpleasant” category generally elicited low ratings of valence. Table 2 shows the mean duration and intensity, in addition to the standard devitation (SD) of the duration and intensity, as reported by Belin et al. for the sounds used in each valence category.

**TABLE 1.** Estimated mean ratings of valence and arousal (on a 100-point scale) of task-irrelevant stimuli used in this study, as reported by Belin et al (2008)

Condition Category	Emotion	Rating of Valence (Mean)	Rating of Arousal (Mean)
Neutral	Neutral	47.5	33.0
Pleasant	Happy	85.5	58.0
	Pleased	70.5	36.0
Unpleasant	Angry	16.5	72.5
	Painful	23.5	66.5
	Sad	19.5	45.0
	Surprised	39.0	71.0

**TABLE 2.** Acoustic data for the three categories of task-irrelevant stimuli, as reported by Belin et al. (2008)

Condition Category	Duration (msec)	Duration SD (msec)	Intensity (dB)	Intensity SD (dB)
Neutral	1066	505	81.1	2.4
Pleasant	1517	529	62.3	9.8
Unpleasant	1276	1316	71.4	7.5

SD indicates the standard deviation of the data across the nine tokens in each category.

### Procedures

Dual-task testing was completed in six conditions, which varied by task-irrelevant vocalization valence (pleasant, neutral, unpleasant) and noise (quiet, noise). Testing in the conditions was blocked such that all the task-irrelevant vocalization valence categories (pleasant, neutral, unpleasant) were tested in either the quiet or the noise condition on the first day of testing. Participants returned to the laboratory for testing on a second day to complete the other block of conditions (noise or quiet; counterbalanced across participants). Within each block of quiet or noise conditions, the order of the emotion categories for the task-irrelevant vocalizations was randomized across participants. For each valence, two lists of 60 words were tested sequentially. Both lists and subjective ratings for one task-irrelevant vocalization valence category were completed before proceeding to the next valence category. Of the eight lists available, two were not used. Each of the six remaining lists was used twice, once in each noise condition (quiet, noise).

The dual-task paradigm required participants to repeat each word (primary task) while simultaneously completing a secondary task. The secondary task was timed word categorization; participants pressed a button if the word they heard could be used as a noun, but withheld their button press if the word could not be used as a noun. Approximately 70% of the words in each list could be classified as nouns. To be consistent with previous literature, all button presses for the secondary task were accepted (Picou & Ricketts 2014; Picou et al. 2016; Huang et al. 2022; Huang et al. 2023). Participants were instructed to press the button before verbally repeating the word; that is, they were instructed to prioritize the speed of the button press over the speed of the word repetition.

During the dual-task testing, task-irrelevant vocalizations were presented immediately before 27 of the 60 monosyllable words (45% of trials in a condition). The words with the task-irrelevant vocalizations before them were randomly selected before study commencement and did not change across participants. For each list, the nine vocalizations in each valence category (pleasant, neutral, unpleasant) occurred three times. They were pseudo-randomly assigned to appear immediately before the 27 words representing the task-irrelevant trials in each list. All participants received the same vocalizations for each list, but the vocalizations were presented in different orders. The time between the onset of task-irrelevant vocalizations and relevant target words varied based on the duration of the task-irrelevant stimulus (mean durations are displayed in Table 2).

The target stimulus began 125 msec following the offset of the task-irrelevant vocalization. The timing between the conclusion of one trial (e.g., participant finished repeating the word or indicated they had not understood it) and the commencement of the next trial (e.g., presentation of either the next target word or a task-irrelevant vocalization that proceeded the next word) depended on the experimenter; they manually advanced the experimental program to move from one trial to the next. Participants were informed only that they might hear task-irrelevant vocalizations but were not otherwise instructed on how to respond to them (e.g., ignore them). The experimenter was not blinded to the presence of the task-irrelevant vocalization or its valence but was blinded to the study hypotheses. Participant word-repetition scoring (i.e., correct/incorrect) was conducted in real time by the experimenter. Word repetition was scored conservatively such that a word had to be repeated precisely to be scored as correct (e.g., no conjugations), although pluralization was acceptable.

All testing took place in a sound-attenuating audiometric test booth (4 × 4.3 × 2.7 m). Target words and task-irrelevant vocalizations were always presented at an average level of 65 dBA. Stimuli were presented from a loudspeaker (unpowered, Tannoy 600, Coatbridge, Scotland) located at 0° and 1.25 m from the participant. The target and task-irrelevant sounds originated from the experimental computer (Dell, Round Rock, Texas, USA) controlled by a custom computer program (Neurobehavioral Systems Presentation v 10, Berkley, California, USA) before being routed through a multichannel sound card (ECHO Layla, Ome, Japan) to an amplifier (Russound Crown, Portsmouth, New Hampshire, USA). Background noise, when present, was presented from four loudspeakers, surrounding the participant, at a distance of 1.25 m and at 45°, 135°, 225°, and 315°. Noise from each loudspeaker had a similar presentation level (~59 dBA) after being routed from the experimental computer using multichannel sound editing software (Adobe Audition, San Jose, California, USA) to the amplifier before reaching the four unpowered loudspeakers (Definitive BP-2X, Carlsbad, California, USA). The overall level of the noise was 65 dBA at the location of the participant, creating a 0 dB SNR. All stimuli were calibrated separately, each using a steady-state noise with the same long-term average spectral shape and level as the concatenated stimuli (without silences between stimuli).

Before experimental testing at each study visit, participants completed a sequence of four practice lists using monosyllabic words not included in the experimental lists. Each practice list also contained 60 words. In the first practice list, participants completed only the secondary task in quiet (timed word categorization). In the second practice list, participants completed the primary and secondary tasks in quiet. In the third list, participants completed both tasks in the presence of the noise. In the final practice list, participants completed only the secondary task in quiet to estimate baseline response time for each participant. Each visit to the laboratory lasted approximately 90 min.

### Data Analysis

Before analysis, word repetition scores were converted to rationalized arcsine units (rau) following the equations found in (Studebaker 1985) to normalize the variance near the extremes. Secondary task response times were calculated relative to the onset of the monosyllable word. Response times that were shorter than 100 msec or more than ±3 SDs from the mean in a list were removed before analysis. Word repetition scores (in rau) and secondary task response times (relative to the onset of the monosyllable word in msec) were analyzed separately. For each outcome, linear mixed-effects models were constructed using the *lmer* function of the *lme4* package (Bates et al. 2015) and analyzed using the *Anova* function of the *car* package (Fox et al. 2012) in R (v 4.3; R Core Team 2023).

For all models, the participant was included as a random intercept. For the response times and word repetition scores, the fixed factors were task-irrelevant vocalization (present, absent), vocalization valence category (pleasant, neutral, unpleasant), and noise condition (quiet, noise). Scores for the “absent” task-irrelevant vocalization type were acquired by examining the scores from trials nested within a test block that did not follow a task-irrelevant stimulus (33 of 60 words in a list), whereas scores for the “present” irrelevant stimulus type were acquired by examining scores for trials that immediately followed a task-irrelevant stimulus (27 of 60 words in a list). That is, all three valence categories included trials that had task-irrelevant vocalizations and trials that did not have task-irrelevant vocalizations. The scores for both types of trials were included in the analyses with a fixed factor of task-irrelevant vocalizations (present or absent). This analytic approach is consistent with previous work (Gustafson et al. 2023; Morgan et al. 2025) and reflects the interpretation of distraction effects as the difference in scores between trials with an irrelevant stimulus being present and absent.

For all models, measures of effect size were calculated using the *anova_stats* function of the *sjstats* package (Lüdecke & Lüdecke 2019); Cohen *f* values were interpreted as 0.10, 0.25, and 0.40, indicating small, medium, and large effects, respectively (Cohen 1988). Statistically significant main effects or interactions were explored using the *emmeans* function of the *emmeans* package (Lenth 2019), adjusting for family-wise error rate (Benjamini & Hochberg 1995). In addition to the inferential statistics, Bayes Factors were calculated for all main effects and interactions using the *lmBF* function of the *BayesFactor* package (Morey & Rouder 2021). Bayes Factors can be useful for evaluating the strength of support of a null or an alternative hypothesis and can complement inferential statistics, especially in the interpretation of nonsignificant statistical results (Brydges & Gaeta 2019). Bayes Factors can be interpreted as moderate, strong, and extreme support for a hypothesis when they are larger than 3, 10, and 100, respectively. The reciprocal of the Bayes Factor can be interpreted as support for the competing hypothesis, so 1/3, 1/10, and 1/100 would be moderate, strong support, and extreme support (Jeffreys 1961). In the case of the present study, Bayes Factors were calculated such that small values (the reciprocal Bayes Factor) support the null hypothesis. In other words, they support that a factor (i.e., task-irrelevant vocalization, vocalization valence category, noise condition) is not significantly related to the outcome variable (i.e., response times, word repetition score).

## RESULTS

### Word Repetition

**Fig. 1.**
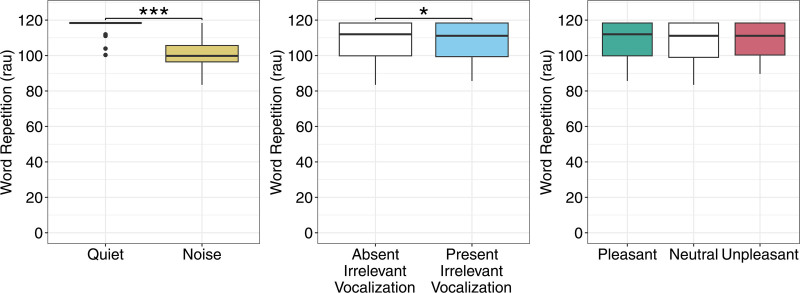
Word repetition scores (rau) in quiet and in noise (left panel), following trials without and with task-irrelevant vocalizations (middle panel), and in lists with vocalizations that were pleasant, neutral, or unpleasant (right panel). Significant differences are indicated by *** (*p* < 0.001) or by * (*p* < 0.05). Boxes represent the 1st to 3rd quartile; solid bars indicate median scores.

Analysis results are displayed in Table 3. Figure 1 displays the main effects of noise, presence of task-irrelevant vocalizations, and vocalization valence on word repetition performance (in rau); the potential interactions are not displayed. In brief, word repetition performance was not related to task-irrelevant vocalization valence category (pleasant, neutral, unpleasant) either as a main effect or in an interaction, as evidenced by large *p* values and Bayes Factors less than 0.10. Conversely, background noise did significantly reduce word repetition performance, as evidenced by a small *p* value and a Bayes Factor larger than 100. The estimated marginal means were 117 and 101 rau in quiet and noise, respectively (*t*[379] = 23.28, *p* < 0.0001).

**TABLE 3.** Analysis results of word repetition scores with main effects and interactions based on model comparisons and Bayes factors (values above 10 provide evidence of the alternative hypothesis; values less than 0.10 provide evidence in support of the null hypothesis)

Term	χ^2^	df	*p*	Cohen *f*	Bayes Factor
Noise condition	541.83	1	<0.00001	1.16	2.75e+75
Valence category	0.52	2	0.769	0.05	0.03
Irrelevant vocalization	4.27	1	0.039	0.06	0.16
Noise condition × Valence category	1.67	2	0.434	0.07	0.08
Noise condition × Irrelevant vocalization	0.56	1	0.456	0.07	0.17
Valence category × Irrelevant vocalization	0.70	2	0.703	0.03	0.10
Noise condition × Valence category × Irrelevant vocalization	0.37	2	0.829	0.05	0.13

As indicated in Table 3, there was a small statistically significant effect of the presence of task-irrelevant vocalizations. For words that immediately followed a task-irrelevant vocalization, word repetition performance was lower (estimated marginal mean = 108 rau) than for words that were not preceded by a task-irrelevant vocalization (estimated marginal mean = 110 rau; *t*[379] = 2.07, *p* = 0.039), suggesting a small distraction effect. However, the Bayes Factor for the effect of the presence of task-irrelevant vocalizations was small (0.16), suggesting moderate support for the null hypothesis (Jeffreys 1961). The combination of a small calculated distraction effect, small Cohen *f*, and a small Bayes Factor suggests the significant effect of task-irrelevant vocalization in Table 3 is specious. These results indicate that essentially only background noise affected word repetition performance.

### Response Times

**Fig. 2.**
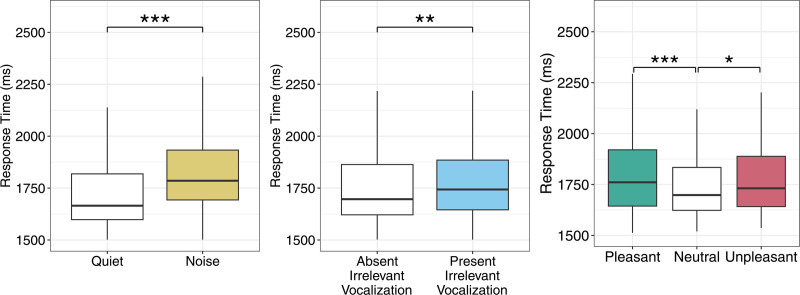
Response times in quiet and in noise (left panel), following trials without and with task-irrelevant vocalizations (middle panel), and in lists with vocalizations that were pleasant, neutral, or unpleasant (right panel). Significant differences are indicated by *** (*p* < 0.001), ** (*p* < 0.01), or * (*p* < 0.05). Boxes represent the 1st to 3rd quartile; solid bars indicate median scores.

Analysis results are displayed in Table 4. In brief, these results indicate significant main effects of noise, vocalization valence, and presence of task-irrelevant vocalization, but no significant interactions (according to the inferential statistics and small Bayes Factors). Figure 2 displays the main effects of noise, presence of task-irrelevant vocalizations, and vocalization valence on response times (in msec); the potential interactions are not displayed.

**TABLE 4.** Analysis results of response times with main effects and interactions based on model comparisons and Bayes factors (values above 10 provide evidence of the alternative hypothesis; values less than 0.10 provide evidence in support of the null hypothesis)

Term	χ^2^	df	*p*	Cohen *f*	Bayes Factor
Noise condition	150.98	1	<0.00001	0.85	1.21e+27
Valence category	14.04	2	<0.0009	0.25	2.63
Irrelevant vocalization	9.31	1	0.002	0.21	3.14
Noise condition × Valence	1.39	2	0.498	0.08	0.09
Noise condition × Irrelevant vocalization	0.004	1	0.950	0.01	0.15
Valence category × Irrelevant vocalization	3.98	2	0.137	0.13	0.29
Noise condition × Valence category × Irrelevant vocalization	0.80	2	0.669	0.07	0.13

Specifically, the addition of background noise slowed secondary task response times (estimated marginal means = 1690 and 1809 msec in quiet and noise, respectively; *t*[380] = −12.29, *p* < 0.0001). In addition, responses were slower to words that followed task-irrelevant vocalizations (estimated marginal mean = 1765 msec) compared with responses that did not follow task-irrelevant vocalizations (estimated marginal mean = 1735 msec, *t*[380] = −3.05, *p* = 0.002). Last, secondary task response times were significantly faster during lists with neutral task-irrelevant vocalizations (estimated marginal mean = 1726 msec) than lists with pleasant task-irrelevant vocalizations (estimated marginal mean = 1769 msec; estimated difference = 43.6 msec; *t*[380] = 3.68, *p* < 0.001) or lists with unpleasant task-irrelevant vocalizations (estimated marginal mean = 1755 msec; estimated difference = 29.3 msec; *t*[380] = 2.47, *p* = 0.04). Response times were not significantly different between lists with pleasant and unpleasant task-irrelevant vocalizations (estimated difference = 14.3 msec, *t*[380] = 1.20, *p* = 0.452). These main effects are also supported by large Bayes Factors, consistent with the inferential statistics.

In summary, these data indicate the expected effect of slowed response times in the presence of background noise. In addition, there was a significant distraction effect; the presence of task-irrelevant vocalizations slowed responses to target words following the task-irrelevant vocalizations. Last, task-irrelevant vocalizations that were pleasant and unpleasant had a general effect of slowing secondary task response times across trials with and without task-irrelevant vocalizations. The presence of the large *p* values, small Cohen *f* values, and small Bayes Factors for the interactions suggests that each of these effects is generally independent of the others. That is, valenced stimuli were not more distracting than neutral stimuli, even though pleasant and unpleasant vocalizations had the effect of generally slowing response times, indicating increased listening effort, during testing within a given condition.

## DISCUSSION

The purpose of this study was to evaluate the effect of task-irrelevant human vocalizations on word repetition performance and behavioral listening effort (secondary task response times) during dual-task testing. Task-irrelevant vocalizations were recordings of humans producing a vowel sound while conveying pleasant, neutral, or unpleasant emotions. Taken together, the results of this study demonstrate that task-irrelevant vocalizations can cause distraction, regardless of their valence category, as evidenced by increased listening effort. In addition, task-irrelevant vocalization valence had a general effect of increasing listening effort. It is interesting that auditory distraction was essentially not evident on the word repetition measure.

### Word Repetition Performance

The conclusion that task-irrelevant vocalizations did not affect word repetition performance is based on the finding that differences in scores were small (~2 rau), a small Cohen *f* effect size (0.07), and a small Bayes factor (0.16). On the basis of previous investigations of auditory distraction, it was hypothesized that the presence of either pleasant or unpleasant task-irrelevant vocalizations would reduce word repetition performance, but only in quiet. This hypothesis was based on the Load Theory of Attention (Lavie & Tsal 1994; Lavie 1995), which states distraction is most likely when the perceptual load of the task is low (i.e., in quiet), compared with when the perceptual load of the task is high (i.e., in noise). However, the results of the present study do not directly support the Load Theory of Attention and are inconsistent with similar work (Gustafson et al. 2023; Morgan et al. 2025). Specifically, the presence of task-irrelevant affective vocalizations had a negligible effect on word repetition performance. Moreover, there was no interaction with background noise condition, suggesting the effect of task-irrelevant vocalization was not present in either quiet or in noise (as evidenced also by small Cohen *f* and Bayes factor).

One potential explanation is that our task was more demanding than the tasks used previously. Previous research has shown reduced distraction effects when task demands increase, causing a shielding from distraction in conditions of high perceptual load due to increased cognitive load (Harmony et al. 2000; Berti & Schröger 2003; Parmentier et al. 2008; SanMiguel et al. 2008). Gustafson et al. (2023) used a single task (word repetition) to derive the behavioral measure of listening effort (verbal response time). They showed ~4.5% decrease in word repetition in the presence of task-irrelevant non-speech sounds (and only in quiet). In the present study, distraction effects on word repetition performance were less than 2 rau. During the present study, participants completed a dual-task paradigm, where word repetition was paired with a challenging secondary task (to judge whether or not the word heard was a noun). In previous studies, this secondary task has been shown to be more challenging than simpler secondary tasks (Picou & Ricketts 2014). Thus, it is possible that, despite being completed in a low perceptual load condition (i.e., with excellent word repetition performance), the cognitive load required by the conditions in the present study was high enough to prohibit word repetition deficits associated with task-irrelevant sounds that have been shown previously (Gustafson et al. 2023). The increased difficulty of the dual-task relative to the single task might account for the negligible effect of task-irrelevant sounds on word repetition performance that was evident in quiet and in noise.

There are other potentially relevant methodological differences that might account for the surprisingly small distraction effects in quiet and in noise in the present study. For example, the present study used speech sounds, which have been shown to interfere more with verbal recall than non-speech sounds (Leist et al. 2025). In addition, Gustafson et al. (2023) presented target speech to one ear and task-irrelevant non-speech sounds to the contralateral ear, whereas in this study, the task-irrelevant vocalizations and target words were collocated from a loudspeaker in front of the listener. Furthermore, in the present study, task-irrelevant vocalizations were present on 45% of trials, and the valence category was blocked within a condition, unlike the less frequent and mixed valence methods used in much of the previous work investigating distraction effects (Most et al. 2005, 2007; Attar et al. 2010; Morgan et al. 2025). Reduced novelty of repeated stimuli could reduce distractibility, as has been shown in the visual (Paul et al. 2016) and auditory domains (Jones & Macken 1993; Roeber et al. 2005). Future work is warranted to explore the potential roles of these methodological variables in the distraction effects of vocalizations.

### Behavioral Listening Effort

On the basis of previous literature (Gustafson et al. 2023; Morgan et al. 2025), it was expected that distraction effects (increased listening effort following trials with task-irrelevant vocalizations) would be evident in quiet and in noise. Consistent with these expectations, distraction effects were evident in the secondary task response times in both quiet and in noise. However, unlike previous work where pleasant and unpleasant task-irrelevant non-speech sounds were more effective distractors than were neutral non-speech sounds (Morgan et al. 2025), in the present study, distraction effects (the differences in response times between trials with and without distractors) were similar in conditions with pleasant, neutral, and unpleasant vocalizations. This similarity in distraction effects across valence conditions is evidenced by the nonsignificant interaction between valence and the presence of task-irrelevant vocalization. The nonsignificant effect also had an associated small Bayes Factor and small Cohen *f* effect size.

This indicates that the valence of the vocalization had a general effect of increasing listening effort (across a whole block) that was not specific to trials with a distractor. The reasons for this are not clear, but it is possible that the distraction effects in the present study are not limited to the trials where the irrelevant stimuli occur. The effects of the task-irrelevant vocalizations might carry over to trials without irrelevant sounds, resulting in general increases in slowed responses overall, rather than slowed responses specifically after an irrelevant sound. Future work is warranted to examine the time course of the distraction effects to evaluate if the increased listening effort for pleasant and unpleasant conditions in the present study is indeed due to carryover distraction effects or another phenomenon. Such work would benefit from a condition with no irrelevant stimuli, rather than interleaving trials with and without irrelevant stimuli.

Note that even though the effect of valence on response times was statistically significant (with a moderate Cohen *f* effect size and moderate Bayes Factor support), the difference between conditions is relatively small compared with the effects previously reported (Morgan et al. 2025). Specifically, in the present study, background noise and the presence of task-irrelevant vocalizations increased response times by 119 and 30 msec, respectively. These are a 7% and 2% increase in response times, relative to the average response times in quiet and when no task-irrelevant vocalizations were present, respectively. Conversely, Morgan et al. (2025) reported effects of background noise and task-irrelevant pleasant non-speech sounds to increase listening effort by 38 and 260 msec, respectively, reflecting a 3% increase in response times as a result of background noise and a 21% increase in response times due to task-irrelevant pleasant, non-speech sounds.

The relative differences between studies (size of distraction effect and valence-specific effects) could be due to methodological task differences. First, the rate of occurrence for task-irrelevant vocalizations was higher in this study than in previous work (45% versus 24%). Previous research has reported reduced distraction effects with rates near 50% (Jankowiak & Berti 2007). Indeed, in studies implementing oddball paradigms, listeners can habituate to the novelty of oddballs when their presentation rates are high (Lammers & Badia 1989; Polich & McIsaac 1994; Debener et al. 2002).

Second, the participants in previous studies were instructed to ignore the task-irrelevant sounds (Gustafson et al. 2023; Morgan et al. 2025), whereas participants in the present study were only alerted to the existence of the task-irrelevant vocalizations; they were not told to attend to or ignore them. It is possible that actively ignoring the irrelevant vocalizations could be more cognitively demanding than simply performing a word repetition task and not focusing on irrelevant vocalizations. This speculation is consistent with existing research in the visual domain, which has shown that, when the participants were cued about the to-be-ignored items, they first briefly attend to irrelevant stimuli before ignoring them (Lahav et al. 2012; Moher & Egeth 2012). This paradox, wherein participants attend to task-irrelevant stimuli before ignoring them, could suggest that participants who have been instructed to ignore task-irrelevant stimuli might be more prone to the distraction effect than participants who have not been instructed to ignore the stimuli. If future work confirms this speculation, there might be implications for handling distracting events in daily life, such as in classrooms.

A final, crucial difference between previous studies and the present study is the use of human vocalizations as the task-irrelevant sounds. As discussed in the introduction, speech sounds and non-speech sounds are processed differently from one another, and literature examining distraction effects for speech versus non-speech sounds has been mixed. Compared with the results of Morgan et al. (2025), listeners in the present study displayed greatly reduced distraction from irrelevant vocalizations in terms of listening effort compared with distraction from irrelevant non-speech sounds. Indeed, reaction times were almost five times longer for distracting stimuli (non-speech sounds) in Morgan et al. than for those in the present study (vocalizations). Recall as well that the present study found only a small effect of distractors on word repetition performance (~2 rau). Thus, despite the differences in methodologies between studies, the results suggest that human vocalizations are, in general, less distracting than non-speech sounds. This may be due to the inherently social nature of human speech sounds, particularly those intended to convey a particular emotion. Natural conversational speech rarely contains speech sounds from only a single individual; listeners frequently use vocal interjections (e.g., “affect bursts,” such as laughter, or “affect emblems,” such as the exclamation “yuck”; Scherer 1994) to indicate their emotional response to what is being said. In addition, overlap between the speech of conversational partners is frequently seen in natural speech corpora (Heldner & Edlund 2010). Thus, it is possible that listeners may expect, to some extent, affective exclamations from other speakers much more than they expect non-speech sounds.

Despite these methodological differences, it is clear that task-irrelevant sounds increase listening effort, whether the sound is a vocalization (present study) or a non-speech sound (Gustafson et al. 2023; Morgan et al. 2025). Thus, although previous research provides inconsistent support for the relative distraction effects of speech and non-speech sounds (Jones et al. 1990; Jones & Macken 1993; Marsh et al. 2009; Reiche et al. 2013), the results of the present study suggest that human vocalizations and non-speech sounds can both be distracting, albeit to different extents. Future work is warranted to examine the relative distractibility of speech and non-speech sounds and to address inconsistencies potentially attributable to the other methodological differences between the aforementioned studies.

## CONCLUSIONS

The primary purpose of this project was to evaluate the effect of task-irrelevant vocalizations on word repetition performance and behavioral listening effort. The results of this study demonstrate that pleasant, neutral, and unpleasant task-irrelevant vocalizations increased behavioral listening effort in quiet and in noise, whereas they had only a small effect on word repetition performance (~2 rau). The valence of the vocalization had no effect on word repetition performance, but did have an effect of increasing listening effort generally. That is, conditions with valenced task-irrelevant vocalizations (pleasant/unpleasant) resulted in more listening effort than did conditions with neutral task-irrelevant vocalizations, rather than having a specific effect of increasing effort only immediately after task-irrelevant vocalizations. This could reflect carryover of distraction to trials without task-irrelevant sounds, which warrants further investigation. Future work is also warranted to disentangle the methodological differences between the present study and previous work examining the effects of task-irrelevant sounds on speech repetition performance and behavioral listening effort. The findings also have implications for listening in complex, real-world environments, where distraction and valenced sounds are likely to co-occur.

## ACKNOWLEDGMENTS

Portions of this project were presented at the Scientific and Technical Conference of the American Auditory Society (Scottsdale, AZ, February 2025).

## Footnotes

* The MAV sounds used in this study, which are labeled as “talker number_emotion” were: 42_happiness, 42_neutral, 45_neutral, 46_neutral, 46_pain, 53_happiness, 53_neutral, 55_happiness, 55_neutral, 55_surprise, 58_happiness, 58_neutral, 58_pain, 59_happiness, 59_sadness, 6_happiness, 6_neutral, 6_pain, 6_surprise, 60_anger, 60_happiness, 60_neutral, 60_pleasure, 60_sadness, 61_happiness, 61_neutral, 61_surprise.
